# Dietary intake in undernourished adults living in Guinea-Bissau; a cross-sectional study

**DOI:** 10.1186/s40795-019-0276-9

**Published:** 2019-02-15

**Authors:** Cecilie Blenstrup Patsche, Frauke Rudolf, Antonio Mateus da Silva Mendes, Idalina da Cunha, Victor Francisco Gomes, Christian Wejse, Charlotte Jeppesen

**Affiliations:** 1 Bandim Health Project, INDEPTH network, Apertado 861 GW-1004 Bissau-Codex, Bissau, Guinea-Bissau; 2 Center for Global Health (GloHAU), Department of Public Health, Aarhus University, Bartholins Allé 2, building 1260, DK-8000 Aarhus C, Denmark; 3 Department of Infectious Diseases, Aarhus University Hospital, Palle Juul-Jensens Blvd 99, DK-8200 Aarhus N, Denmark; 4 Section for Health Promotion and Health Services, Department of Public Health, Aarhus University, Bartholins Allé 2, building 1260, DK-8000 Aarhus C, Denmark; 5 The Danish Diabetes Academy, Odense University Hospital, Sdr Blvd 29, entrance 112, 3rd floor, DK-5000 Odense C, Denmark; 6 University College Copenhagen, Nursing Education, Carlsbergvej 14, 3400 Hilleroed, Denmark

**Keywords:** Dietary intake, Nutrition assessment, West Africa, Undernutrition, 24-h dietary recall, Guinea-Bissau

## Abstract

**Background:**

Data on dietary intake in Guinea-Bissau is limited. The main purpose of this study was to compare mean daily energy intake (EI) with mean daily energy expenditure (EE) for a moderately active lifestyle in health-seeking individuals with a body mass index < 20.0 kg/m^2^. Furthermore, dietary composition was evaluated by estimates of macronutrient energy distribution, individual dietary diversity scores, and the identification of frequently consumed food items, stratified by sex.

**Methods:**

A cross-sectional dietary survey was conducted at the suburban health- and demographic surveillance site in Guinea-Bissau, West Africa, from May 2014–February 2015. Each participant answered one interviewer-administered 24-h dietary recall at baseline, from which dietary intake was assessed. Differences in dietary intake between men and women were analysed using the *X*^2^ test or Fisher’s Exact test for categorical outcomes, and the Student’s t-test for continuous variables.

**Results:**

Forty-three men and forty-eight women were included. Mean EI for men was 6326 kJ/d (sd 2104) and for women 6822 kJ/d (sd 2210). Mean EE for a moderately active lifestyle with a physical activity level of 1.75 was 10,479 kJ/d (sd 658) for men and 8729 kJ/d (sd 731) for women. Both men and women had a significantly lower mean EI compared with mean EE (*p*-values both < 0.001). Dietary diversity was low with a score of 3.5 (sd 1.0) for men and 4.0 (sd 1.3) for women, *p-*value 0.046. Macronutrient energy was distributed as 66% (sd 11) carbohydrate, 15% (sd 5) protein, and 19% (sd 9) fat, with no significant difference in distribution between men and women. Consumption of starchy and sugary carbohydrates accounted for two thirds of mean EI. Cereals were the main source of protein, in place of animal protein.

**Conclusions:**

Both men and women in this study had low mean EI compared with mean EE for a moderately active lifestyle. Dietary intake was characterized by a seemingly low dietary diversity and imbalanced macronutrient energy distribution, comprising insufficient fat intake and excess carbohydrate intake. Cereals were the main source of protein.

**Trial registration:**

PACTR2009110001673419. Registered 22 Oct 2009.

## Background

The United Nations Decade of Action on Nutrition (2016–2025) began with a glooming message that the number of undernourished people in the world had increased from 777 million in 2015 to 815 million in 2016 [1]. Undernutrition can be caused by a number of factors including medical conditions, infectious diseases, and insufficient energy intake. The latter may be accentuated by food insecurity prompted by prolonged conflicts, political instability and food unavailability, especially in developing countries [1].

In the West African country Guinea-Bissau, the proportion of the total population being undernourished (i.e. in a “condition in which an individual’s habitual food consumption is insufficient to provide the amount of dietary energy required to maintain a normal, active, healthy life” [1]) was estimated to be 28.3% in 2014–2016; an unfortunate increase from 24.9% a decade earlier [1].

Information on dietary intake is essential in order to develop focused nutrition strategies aimed at improving nutritional status and health of undernourished individuals. There are no previous studies on dietary intake and diet composition of adults from Guinea-Bissau [2], and only very limited, recent data from other West African countries [3–5].

The main purpose of this study was to compare mean daily energy intake (EI) with mean daily energy expenditure (EE) for a moderately active lifestyle in health-seeking individuals with a body mass index (BMI) < 20.0 kg/m^2^ living in Guinea-Bissau. Furthermore, dietary composition was evaluated by estimates of macronutrient energy distribution, individual dietary diversity score, and the identification of frequently consumed food items, stratified by sex.

## Methods

### Study design and setting

The present cross-sectional dietary survey was part of a randomized controlled pilot trial evaluating the use of nutritional support to lower mortality and reduce prevalence of undernutrition among patients with presumed pulmonary tuberculosis (unpublished observations, principal investigator Frauke Rudolf, registration number PACTR2009110001673419). For the purpose of this dietary survey, all participants diagnosed with tuberculosis were excluded. Only information obtained at baseline interviews, conducted from May 2014 to February 2015, were reviewed in this study.

The trial was conducted at the health- and demographic surveillance site, the Bandim Health Project [6], located in Bissau, the capital of Guinea-Bissau. The Bandim Health Project study area hosts a population of approximately 102,000 residing in six defined suburbs. The capital Bissau is a coastal city, and residents have easier access to fresh fish and vegetables at food markets compared with the rest of the country. Guinea-Bissau is one of the poorest countries in the world. The country economy is dependent on small-scale agriculture and fishing, cashew nut export and foreign aid, and is highly impacted by prolonged political instability.

### Participants

Participants were recruited from the three health centres situated in the Bandim Health Project’s study area, when seeking medical care. All health-seeking individuals who presented with either cough, sputum production, or weight loss, regardless of the duration of symptoms, were eligible for inclusion if meeting following inclusion criteria: Age ≥ 15 years, BMI < 20.0 kg/m^2^, and residence in the study area. For the purpose of this dietary survey, participants diagnosed with tuberculosis (either by sputum smear microscopy or GeneXpert MTB/RIF) were excluded. Self-reported symptoms of disease and sociodemographic characteristics were recorded, and anthropometric data were measured.

### Dietary assessment

Each participant answered one interviewer-administered 24-h dietary recall (24HR) at baseline, from which dietary intake was assessed. Data were sampled daily including weekends, thus all days were represented at group level. Rainy season spans from June through October, and seasonal fruit and vegetables span over three months, thus the 10-month inclusion period from May through February accounted for seasonal variability at group level.

Two local field assistants were appointed to conduct the interviews. Before commencement of the study, the field assistants practiced their interview skills on four volunteers with a BMI < 20.0 kg/m^2^. The assistants supervised each other during those interviews, to help establish a consensus of interview method among them. The field assistants were trained to use local utensils (bowls, plates, cups and spoons) to assist participants in estimating portion sizes. They were also trained to probe for details including time of consumption of food items, method of preparation, amounts consumed, condiments added, seasonal variations to recipes and, when applicable, brand names.

Local recipes were gathered from four chosen, independent women living in different suburbs of Bissau. The base recipes supplied by the four women were similar, and it was agreed that average base recipes for local dishes could be calculated from those. Participants were asked to specify any seasonal vegetables added to consumed dishes, which were then accounted for when calculating individual EI. All 24HRs were checked for misreporting and invalid amounts/portion sizes thrice weekly.

No food composition database for Guinea-Bissau was available, and one was therefore compiled using primarily the West African Food Composition Table by the Food and Agriculture Organization of the United Nations (FAO) [7]. Missing food items were identified in the Food Composition Table for use in Africa also by FAO [8], and where relevant, brand labels were entered into the database. All food items were translated into energy values by multiplying by energy content factors: 17 kJ/g for carbohydrate and protein, and 37 kJ/g for fat [9]. None of the participants reported consuming alcohol. Over-reporters were classified as EI > 16,736 kJ/d for men and EI > 14,644 kJ/d for women [10]. Very low EI was not reason for exclusion because it could be expected of participants with BMI < 20 kg/m^2^. Macronutrient energy distribution in percentage (E%) was calculated as the consumed amount in g multiplied by the respective energy content factor divided by daily energy intake. According to World Health Organization and Institute of Medicine, recommended energy distribution is: 45–65 E% carbohydrate, 10–35 E% protein, and 20–35 E% fat [11–13].

Group mean basal metabolic rate (kJ/d) was calculated using the FAO/World Health Organization/United Nations University equation [14], as a recent validation study proved it best for adult underweight patients [15]. Group mean EE (kJ/d) for a moderately active lifestyle (physical activity level (PAL) of 1.75) were calculated by multiplying basal metabolic rate with PAL [16]. EE for a moderately active lifestyle was chosen as the reference, because it was considered an average PAL for the studied population. Some participants would have had a more sedentary lifestyle due to unemployment, underlying medical conditions etc., whereas others would have had a much more active lifestyle due to hard physical labour both domestically and at work depending on season.

The Women’s Dietary Diversity Score was used to evaluate individual dietary diversity. The score was developed to assess micronutrient adequacy of diets of women of reproductive age, but FAO guidelines suggest this score can be applied to other age- and sex groups [17]. The score reflects micronutrient inadequacy of the diet, and is based on 9 tailored food groups: “Starchy staples”, “Dark green leafy vegetables”, “Other vitamin A rich fruits and vegetables”, “Other fruits and vegetables”, “Organ meat”, “Meat and fish”, “Eggs”, “Legumes, nuts and seeds”, and “Milk and milk products”. Food quantities < 15 g was not included in the score. A low diet diversity is indicated by a score < 4 (maximum score is 9).

### Anthropometry

Body weight was measured to the nearest 0.1 kg using a digital bathroom scale (IDA Foundation, The Netherlands), with the participant barefooted and wearing minimal clothing. Height was measured to the nearest cm, with the participant barefooted, using a roll-up tape. Mid upper-arm circumference was measured at the midpoint between the acromion and olecranon on the non-dominant arm, with the arm hanging loosely, using a non-stretchable tape (TALC, Herts, UK) measuring to the nearest 2 mm. Classification of BMI groups was according to World Health Organization guidelines [18].

### Study size

Post-hoc power calculations with a 0.05 significance level, using data from Table 2, were performed. A sample of 43 men can detect a difference between mean EI of 6326 kJ/d (sd 2104) and mean EE for a moderately active lifestyle with PAL of 1.75 of 10,479 kJ/d (sd 658) with 100% power. A sample of 48 women can detect a difference between mean EI of 6822 kJ/d (sd 2210) and mean EE for a moderately active lifestyle with PAL of 1.75 of 8729 kJ/d (sd 731) with 100% power.

### Statistical analyses

Data were entered in Excel 2010 (Microsoft Corporation, Redmond, WA, USA), and all statistical analyses were performed using STATA SE 11.2 (STATA Corporation, College Station, TX, USA). Differences between mean EI and mean EE as well as differences between men and women were analysed as follows: Histograms and QQ-plots were used to evaluate if data were normally distributed. Continuous variables with normal distribution (reported as means with standard deviation) were compared using the Student’s t-test. Differences in categorical outcomes were assessed using the *X*^2^ test, or Fisher’s Exact test if sub cell count was less than five observations. A two-sided *p*-value of ≤0.05 was considered statistically significant. Sensitivity analyses were not performed due to the low sample size.

## Results

### Participants

One hundred and eight participants were included in the study. Twelve participants diagnosed with tuberculosis were excluded. A further five participants were excluded based on dietary recall responses: one questionnaire was deemed invalid due to suspected misreporting, three were over-reporters regarding energy intake and one reported having a very atypical day with an energy distribution of 11 E% carbohydrate and 70 E% fat. In total, data from forty-three (47%) men and forty-eight women were obtained. Descriptive characteristics of the study population stratified by sex is presented in Table 1. No differences were found between men and women in distribution of age, ethnicity or self-reported disease symptoms, but men did have a higher body weight and mid upper-arm circumference compared with women. Half (51%) of men and two-thirds (65%) of women were undernourished (BMI < 18.5 kg/m^2^), of which 7% of men and 13% of women were severely undernourished (BMI < 16 kg/m^2^).

**Table 1:** Descriptive characteristics for the adult Bissau-Guinean study population (*n* = 91) stratified by sex

	Total (*n* = 91)	Men (*n* = 43)	Women (*n* = 48)	
Characteristic	*n* (%)	*n* (%)	*n* (%)	*p*-value^a^
Age, *n* (%)				
15–24 years	35 (38)	19 (44)	16 (33)	
25–34 years	22 (24)	11 (26)	11 (23)	
35–44 years	8 (9)	1 (2)	7 (15)	
45–54 years	6 (7)	3 (7)	3 (6)	
55- years	20 (22)	9 (21)	11 (23)	0.319
Ethnicity, *n* (%)				
Balanta	6 (7)	3 (7)	3 (6)	
Fula	5 (6)	1 (2)	4 (9)	
Mancanha	5 (6)	1 (2)	4 (9)	
Mandinga	6 (7)	3 (7)	3 (6)	
Manjaco	19 (21)	8 (19)	11 (24)	
Pepel	36 (41)	19 (46)	17 (37)	
Other^b^	11 (12)	7 (17)	4 (9)	0.607
BMI, *n* (%)				
< 16.0 (kg/m^2^)	9 (10)	3 (7)	6 (13)	
16.0–18.49 (kg/m^2^)	44 (48)	19 (44)	25 (52)	
18.5–19.99 (kg/m^2^)	38 (42)	21 (49)	17 (35)	0.373
Self-reported weight loss, *n* (%)	68 (75)	32 (74)	36 (75)	0.949
Loss of appetite, *n* (%)	52 (57)	21 (49)	31 (65)	0.130
Fatigue, *n* (%)	59 (65)	27 (63)	32 (67)	0.699
Diarrhoea, *n* (%)	10 (11)	6 (14)	4 (8)	0.508
	mean (sd)	mean (sd)	mean (sd)	
Body weight (kg)	50.2 (7.0)	54.0 (5.9)	46.8 (6.1)	< 0.001
MUAC^c^ (mm)	267 (29)	274 (28)	260 (29)	0.029

*n* sample size, *BMI* body mass index, *MUAC* mid upper arm circumference

^a^*p*-value for difference between men and women

^b^Other ethnicities include smaller Bissau-Guinean and non-Bissau-Guinean groups. Three participants had missing data on ethnic status

^c^Seven participants had missing data on MUAC

### Dietary intake

The majority (73%) of participants reported that the past 24 h had been a typical day. Main meals consisted of either rice, fish or meat, and sauce, or bread/sandwiches, and were consumed at times corresponding to breakfast, lunch and dinner. Snacks were sometimes consumed in place of main meals but were considered meals. Eighteen percent had consumed two meals/d, 75% had consumed three meals/d, and the remaining participants had consumed either one or four meals/d. Only 15 % reported snacking. Meal patterns did not differ significantly between men and women.

Table 2 presents daily energy- and macronutrient intake and dietary diversity score, stratified by sex. Both men and women had a significantly lower mean EI compared with mean EE for a moderately active lifestyle (*p*-values for both men and women were < 0.001). Table 3 lists the five single food items contributing most to energy- and macronutrient intake for men and women, depicting the most commonly consumed food groups in the studied population.

**Table 2:** Daily energy- and macronutrient intake stratified by sex

	Total (*n* = 91)	Men (*n* = 43)	Women (*n* = 48)	
	Mean (sd)	Mean (sd)	Mean (sd)	*p*-value^a^
Daily energy intake (EI) (kJ/d)	6588 (2163)	6326 (2104)	6822 (2210)	0.277
Daily energy expenditure (EE) (kJ/d)	9556 (1119)	10,479 (658)	8729 (731)	< 0.001^b^
Total carbohydrate intake (g/d)	255 (91)	250 (80)	259 (99)	0.629
Total protein intake (g/d)	56 (25)	52 (27)	59 (23)	0.234
Total fat intake (g/d)	35 (23)	31 (22)	38 (24)	0.154
Dietary diversity score	3.7 (1.2)	3.5 (1.0)	4.0 (1.3)	0.046
	*n* (%)	*n* (%)	*n* (%)	
E% carbohydrate	66 (11)	68 (10)	64 (12)	0.093
E% protein	15 (5)	14 (5)	15 (6)	0.369
E% fat	19 (9)	17 (8)	20 (10)	0.092

*n* sample size, *E%* energy percentage

^a^*p*-value for difference between men and women

^b^*p*-value represent difference between daily mean EI and mean EE, and is the same for both men and women

**Table 3:** Top five single food items contributing to energy- and macronutrient intake divided by sex

Men (*n* = 43)				Women (*n* = 48)			
		% of mean EI	Cumulative % of mean EI			% of mean EI	Cumulative % of mean EI
Total energy				Total energy			
1)	White rice	40.4	40.4	1)	White rice	38.8	38.8
2)	Bread^a^	14.9	55.3	2)	Bread^a^	14.1	52.9
3)	Sugar^b^	9.0	64.3	3)	Sugar^b^	7.8	60.7
4)	Powder milk	3.3	67.6	4)	Powder milk	3.4	64.1
5)	Olive oil	3.2	70.8	5)	Mango	2.9	67.0
Carbohydrate				Carbohydrate			
1)	White rice	54.6	54.6	1)	White rice	54.8	54.8
2)	Bread^a^	17.1	71.7	2)	Bread^a^	17.2	72.0
3)	Sugar^b^	12.1	83.8	3)	Sugar^b^	11.6	83.6
4)	Spaghetti	3.8	87.6	4)	Mango	4.0	87.6
5)	Onion	2.0	89.6	5)	Powder milk	2.9	90.5
Protein				Protein			
1)	White rice	23.6	23.6	1)	White rice	22.8	22.8
2)	Bread^a^	15.7	39.3	2)	Bread^a^	13.5	36.3
3)	Bonga Shad^c^ (*Ethmalosa fimbriata*)	10.9	50.2	3)	Bonga Shad^c^ (*Ethmalosa fimbriata*)	7.3	43.6
4)	Mullet^c^ (*Mugil spp.*)	6.8	57.0	4)	Beef	7.1	50.7
5)	Catfish^c^ (*Synodontis spp.*)	4.9	61.9	5)	Seabream Steenbras^c^ (*Lithognathus aureti*)	6.6	57.3
Fat				Fat			
1)	Olive oil	20.4	20.4	1)	Olive oil	15.4	15.4
2)	Powder milk	8.1	28.5	2)	Red palm oil	9.1	24.5
3)	Bread^a^	7.4	35.9	3)	Bread^a^	6.5	31.0
4)	Butter spread^d^	6.7	42.6	4)	Beef	6.5	37.5
5)	Peanuts	6.1	48.7	5)	Powder milk	6.4	43.9

*EI* energy intake

^a^White whey

^b^Sucrose

^c^Types of fish common in the West African diet

^d^Butter spread consists of butter mixed with canola oil

## Discussion

Both men and women in this study had low mean EI compared with mean EE for a moderately active lifestyle, suggesting an inadequate nutrition intake. The dietary intake was characterized by an imbalanced macronutrient energy distribution; carbohydrate contribution exceeded recommended intake and fat contribution did not reach the minimum recommended intake. Consumption of starchy and sugary carbohydrates accounted for two thirds of mean EI. Protein intake was within the recommended range; however, cereals were the main source of protein in place of animal protein. Furthermore, dietary diversity was low, indicating micronutrient deficiency. A large number of participants reported being fatigued and having lost both appetite and weight; symptoms that could both influence dietary intake, but also be a result of a poor and monotone diet.

There are no previous studies on dietary intake from Guinea-Bissau and only a very limited number of studies from other West African countries [3–5, 19]. Countries of West African origin are different from one another with regards to economy and food availability, and studies from Senegal and Benin represent the only comparable data material at this point. A study from Senegal [4] (published in 2009), using similar methodology as the present study, showed great similarities between the Senegalese and the Bissau-Guinean diet, though the Senegalese diet entailed a greater variety of cereals and animal protein. In a study of 200 randomly selected adults from Benin [5] (published in 2009), grains, fish, vegetables, oils and bread and pasta were the most commonly consumed food items. Macronutrient distribution for the traditional diet was 15.5% fat, 5.2% saturated fat, 5% sugar and 12.7% protein, with a mean EI of 9226 kJ/d (sd 1966). The macronutrient distribution was thus similar to the observed distribution in the present study. Mean EI was considerably higher than in the present study, which could be explained by the fact that overweight adults were included in the study from Benin. Both Senegal and Benin have a somewhat better country economy compared with Guinea-Bissau, and an assumed lower food insecurity due to easier access to food and higher educational and socio-economic status [19, 20]. This is reflected in the different energy intake and dietary patterns observed in the studies. The low mean EI observed in the present study does therefore not only seem to be related to the health and nutritional status of participants, but also seem to be influenced by the present state of food insecurity in Guinea-Bissau.

A great effort was invested in ensuring reliable measurements of dietary intake: quality checks were performed thrice weekly and appropriate regional, though not national, food composition databases were used to ensure accurate nutritional composition of food items. Only two local field assistants administered the 24HR, limiting interviewer bias. They both had good cultural knowledge and knowledge about food habits. Local, recognizable utensils were applied as a visual aid. Data were sampled throughout the entire week, with every day represented, in order to limit day-to-day variation at group level. Data were collected throughout a course of ten months to account for all seasonal dietary variations on a group level. A 24HR was chosen because it was minimally invasive and demanding for participants, required no literacy skills, and would give a good overview of dietary composition, to serve as a good basis for further nutrition related research in Guinea-Bissau. An almost even number of men and women were included from a great age span to permit generalizability. Despite the small sample size, dietary patterns can still be described in a monotonous diet such as the reported Bissau-Guinean diet.

The dietary intake calculated from the 24HR was not compared to and validated against dietary intake assessed using other dietary recall methods. Only one 24HR was conducted and because of this, the within-person day-to-day variation of dietary intake was indeterminable. The distribution of dietary intake could not be assessed well, and only population mean EI and group mean EE could be compared. The comparison of mean EI and mean EE represent a one-day snapshot only. Data on actual PAL would have improved the comparison of mean EI with mean EE. Additionally, dietary fibres (8 kJ/g) were not taking into account when calculating EI, resulting in underestimation of EI. The study population was a selective group of health-seeking individuals with a low BMI, and both wealth-, health- and nutritional status are all factors that influence health seeking behaviour, and thus the composition of this study population. Under- and over-reporting are major bias to be considered. Increasing BMI is considered a key predictor of increasing underreporting in Western populations, though this association in African settings is uncertain [21–24]. Contrary cultural aspects may be influential in this context: Poor nutritional status is often a stigma and participants may deliberately overestimate food intake to cover their embarrassment of either poverty or disease. On the other hand, participants may fear losing food aid if reporting a large dietary intake, and hence deliberately underestimate food intake. Other factors influencing underreporting could be inaccuracy in reporting food portions, memory disturbance, and psychosocial and behavioural factors [23]. Knowledge of dietary practices of men may be limited by poor cooking skills, in contrast to women, who are responsible for cooking for the entire family and thus familiar with household measurements. In Bissau-Guinean culture, family members and visitors often eat from a shared bowl or plate, which complicates portion size estimations. Data on socio-economic status were unavailable. The sample size was deemed too small to perform sensitivity analyses, such as stratifying results in BMI groups, or participants reporting having a typical vs atypical day.

To gain representative data from the general population or a specific patient group, further research is needed in urban, suburban and rural areas, and of larger study populations, regardless of BMI status. In spite of limited generalizability, data from this study is the first to show the nutritional contributors of the diet in a Bissau-Guinean population and may serve as a basis for further dietary data collection in this population.

## Conclusion

Both men and women in this study had low mean EI compared with mean EE for a moderately active lifestyle. Dietary intake was characterized by a seemingly low dietary diversity and imbalanced macronutrient energy distribution, comprising insufficient fat intake and excess carbohydrate intake. Cereals were the main source of protein, in place of animal protein. The identification of dietary deficiencies provides guidance for future nutrition modifications to improve nutritional status of undernourished adults in Guinea-Bissau.

## Abbreviations

24HR: 24-h dietary recall
BMI: Body mass index
E%: Energy percentage
EE: Energy expenditure
EI: Energy intake
FAO: Food and Agriculture Organization of the United Nations
PAL: Physical activity level

## References

[1] FAO, IFAD, UNICEF, WFP, WHO (2017). The State of Food Security and Nutrition in the World 2017. Building resilience for peace and food security.

[2] Wejse C, Olesen R, Rabna P, Kaestel P, Gustafson P, Aaby P (2007). Serum 25-hydroxyvitamin D in a West African population of tuberculosis patients and unmatched healthy controls. Am J Clin Nutr.

[3] Vila-Real C, Pimenta-Martins A, Gomes AM, Pinto E, Maina NH (2018). How dietary intake has been assessed in African countries? A systematic review. Crit Rev Food Sci Nutr.

[4] Anderson CA, Bellamy S, Figures M, Zeigler-Johnson C, Jalloh M, Spangler E, et al. Dietary intake of Senegalese adults. Nutr J. 2010;9:7. 10.1186/1475-2891-9-7. 10.1186/1475-2891-9-7 PMC2834576 20167099

[5] Sodjinou R, Agueh V, Fayomi B, Delisle H (2009). Dietary patterns of urban adults in Benin: relationship with overall diet quality and socio-demographic characteristics. Eur J Clin Nutr.

[6] The Bandim Health Project. Available from: www.bandim.org. Accessed 17 Nov 2018.

[7] FAO. West African Food Composition Table. 2012. Available from: http://www.fao.org/infoods/infoods/tables-and-databases/africa/en/. Accessed 17 Nov 2018.

[8] FAO. Food Composition Table for use in Africa 1968. Available from: http://www.fao.org/docrep/003/X6877E/X6877E00.htm. Accessed 17 Nov 2018.

[9] Nordic Council of Ministers. Nordic Nutrition Recommendations 2012. Integrating nutrition and physical activity. Available from: https://www.norden.org/da/node/7832. Accessed 17 Nov 2018.

[10] Willet W. Issues in analysis and presentation of dietary data. In: Willet W. Nutritional Epidemiology. 3rd ed. New York: Oxford University Press; 2013.

[11] FAO, WHO, UNU. Protein and amino acid requirements in human nutrition*.* Report of a joint FAO/WHO/UNU expert consultation 2007. Available from: http://apps.who.int/iris/bitstream/10665/43411/1/WHO_TRS_935_eng.pdf?ua=1. Accessed 17 Nov 2018.

[12] Institute of Medicine. Dietary reference intakes for energy, carbohydrate, fiber, fat, fatty acids, cholesterol, protein, and Amino Acids 2002. Available from: https://iom.nationalacademies.org/Reports/2002/Dietary-Reference-Intakes-for-Energy-Carbohydrate-Fiber-Fat-Fatty-Acids-Cholesterol-Protein-and-Amino-Acids.aspx. Accessed 17 Nov 2018. 10.1016/s0002-8223(02)90346-9 12449285

[13] FAO, WHO. Interim summary of conclusions and dietary recommendations on Total fat & Fatty Acids. 2008. Available from: http://www.fao.org/ag/agn/nutrition/docs/Fats%20and%20Fatty%20Acids%20Summary.pdf. Accessed 17 Nov 2018.

[14] FAO, WHO, UNU. Energy and protein requirements*.* WHO technical report series*.* Geneva*;* 1985. Available from: http://www.fao.org/docrep/003/aa040e/AA040E15.htm#an1. Accessed 17 Nov 2018.

[15] Weijs PJM, Kruizenga HM, van Dijk AE, van der Meij BS, Langius JAE, Knol DL (2008). Validation of predictive equations for resting energy expenditure in adult outpatients and inpatients. Clin Nutr.

[16] FAO. Human energy requirements*.* Report of a Joint FAO/WHO/UNU Expert Consultation. 2001. Available from: http://www.fao.org/docrep/007/y5686e/y5686e00.htm#Contents. Accessed 17 Nov 2018.

[17] FAO. Guidelines for measuring household and individual dietary Diversity 2011. Available from: http://www.fao.org/fileadmin/user_upload/wa_workshop/docs/FAO-guidelines-dietary-diversity2011.pdf. Accessed 17 Nov 2018.

[18] WHO. Management of severe malnutrition: A manual for physicians and other senior health workers. 1999. Available from: http://apps.who.int/iris/bitstream/10665/41999/1/a57361.pdf?ua=1&ua=1. Accessed 17 Nov 2018.

[19] FAO. Food Security and Nutrition Trends in West Africa - Challenges and the Way Forward. Rome: FAO. Available from: ftp://ftp.fao.org/es/esn/nutrition/ouagafinal.pdf. Accessed 17 Nov 2018.

[20] FAO. Food security indicators. 2016. Available from: http://www.fao.org/economic/ess/ess-fs/ess-fadata/en/. Accessed 17 Nov 2018.

[21] Harrison GG, Galal OM, Ibrahim N, Khorshid A, Stormer A, Leslie J (2000). Underreporting of food intake by dietary recall is not universal: a comparison of data from Egyptian and American women. J Nutr.

[22] Johansson L, Solvoll K, Bjørneboe G-A, Drevon CA (1998). Under- and overreporting of energy intake related to weight status and lifestyle in a nationwide sample. Am J Clin Nutr.

[23] Mennen LI, Jackson M, Cade J, Mbanya JC, Lafay L, Sharma S (2000). Underreporting of energy intake in four populations of African origin. Int J Obes.

[24] Orcholski L, Luke A, Plange-Rhule J, Bovet P, Forrester TE, Lambert EV (2015). Under-reporting of dietary energy intake in five populations of the African diaspora. Br J Nutr.

